# Primary Orbital Mucinous Adenocarcinoma: A Malignancy With Rapid Metastasis

**DOI:** 10.7759/cureus.88904

**Published:** 2025-07-28

**Authors:** Víctor Manuel Asensio-Sánchez, Jorge Rodriguez-Espinosa, Laura De Juan-Hernández, Alejandra Vela Martín, María Elena García-Lagarto

**Affiliations:** 1 Ophthalmology, Hospital Clínico Universitario, Valladolid, ESP; 2 Radiology, Hospital Clínico Universitario, Valladolid, ESP; 3 Pathology and Laboratory Medicine, Hospital Clínico Universitario, Valladolid, ESP

**Keywords:** aggressive orbital tumor, cytokeratin 7, immunohistochemistry, metastatic workup, orbit, orbital biopsy, orbital mass, primary mucinous adenocarcinoma, proptosis, signet ring cells

## Abstract

Primary mucinous adenocarcinoma of the orbit is an exceptionally rare epithelial malignancy that closely mimics metastatic disease both clinically and radiologically, often delaying definitive diagnosis. We report the case of a 69-year-old man who presented with progressive ocular pain, axial proptosis, and a well-circumscribed orbital mass on imaging. Histopathological evaluation following incisional biopsy revealed a mucinous adenocarcinoma with signet ring morphology. Immunohistochemical analysis demonstrated strong cytokeratin 7 (CK7) positivity and weak CK20 expression, favoring a primary orbital origin. Comprehensive systemic evaluation failed to reveal a primary tumor elsewhere, supporting the diagnosis of a primary orbital neoplasm. Despite early intervention, the tumor exhibited rapid metastatic progression, and the patient died within six weeks of diagnosis. This case highlights the aggressive clinical course and diagnostic complexity of primary orbital mucinous adenocarcinoma, underscoring the need for high clinical suspicion, prompt biopsy, and a multidisciplinary approach, although prognosis remains dismal.

## Introduction

Primary mucinous adenocarcinoma is an uncommon epithelial malignancy that typically originates in mucin-secreting tissues, including the breast, gastrointestinal tract, thyroid, and cutaneous adnexa [1,2]. When arising from the skin, it exhibits a notable predilection for the periocular region, particularly the eyelids [3]. Although periocular presentations are more frequently described, primary involvement of the orbit is exceedingly rare. Most reported orbital cases correspond to metastatic disease rather than primary tumors [4-10].

The extreme rarity of primary mucinous adenocarcinoma of the orbit presents a considerable diagnostic challenge. Clinically and radiographically, these lesions often mimic more common orbital entities such as metastases, lymphoproliferative disorders, or benign epithelial neoplasms. The absence of pathognomonic imaging features further complicates timely recognition, often resulting in delayed diagnosis and management. Histopathological examination combined with immunohistochemical profiling is critical for establishing the diagnosis and differentiating primary orbital tumors from secondary involvement [8,9].

We present a rare case of primary mucinous adenocarcinoma of the orbit in a 69-year-old man, characterized by rapid progression and fatal outcome. This report emphasizes the importance of early biopsy and comprehensive systemic evaluation in the assessment of atypical orbital masses.

## Case presentation

A 69-year-old man with a history of pulmonary fibrosis secondary to chronic tobacco use presented to our Ophthalmology Department with progressive left ocular pain and axial proptosis of several weeks' duration. He denied diplopia, periorbital swelling, or systemic symptoms.

**Figure 1:**
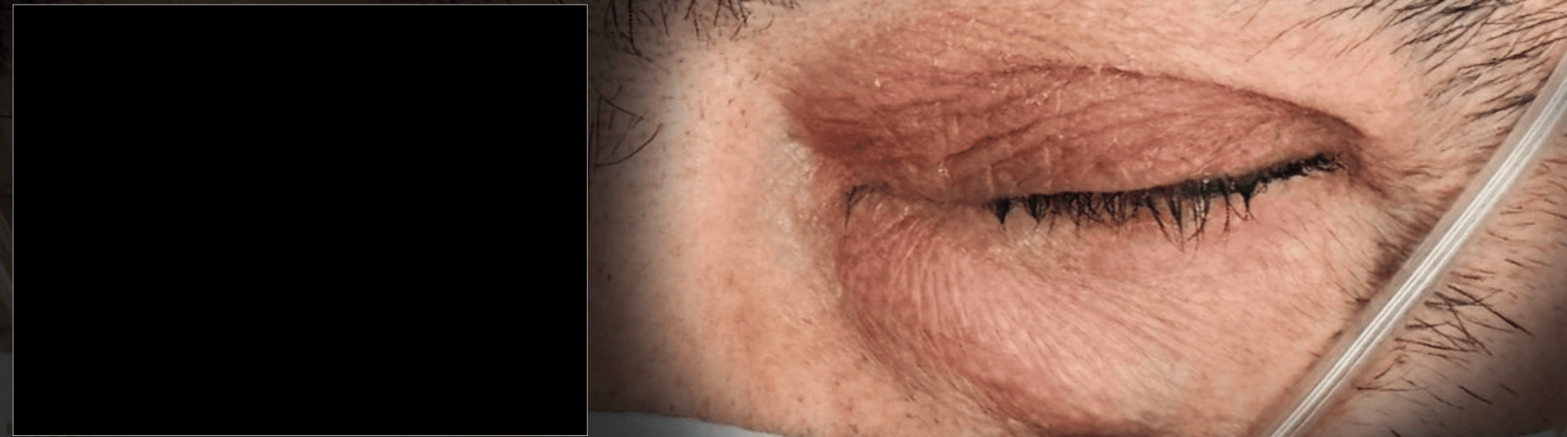
Axial proptosis of the left eye with slight inferior displacement of the globe. Extraocular motility was restricted in abduction, and marked upper eyelid ptosis was observed.

Ophthalmologic examination revealed marked axial proptosis of the left eye with mild inferior displacement of the globe. Extraocular motility was limited in abduction, and evident upper eyelid ptosis was noted (Figure 1). Best-corrected visual acuity was 20/20 in the right eye and no light perception in the left eye. There was no relative afferent pupillary defect. Funduscopy showed a normal optic disc in both eyes, and intraocular pressure was 16 mmHg bilaterally.

**Figure 2:**
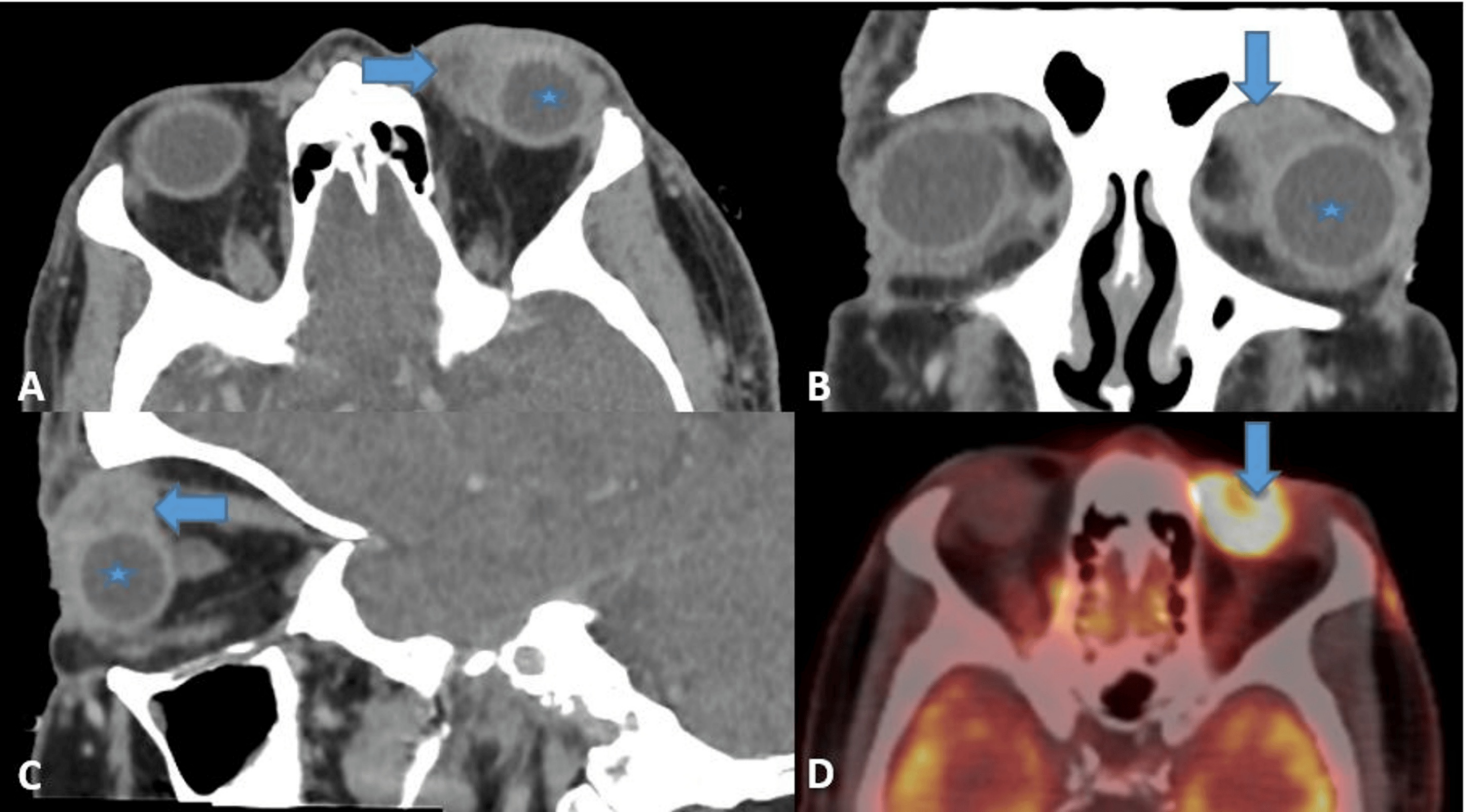
Imaging findings. Contrast-enhanced computed tomography of the orbits with soft tissue reconstruction: axial view (A), coronal view (B), sagittal view (C). Axial view of 18F-FDG PET-CT scan (D). A space-occupying lesion with ill-defined margins (arrows) is observed in the superomedial angle of the left orbit, showing mild peripheral enhancement. The globe is not involved (*). The lesion is hypermetabolic on PET-CT, with a maximum standardized uptake value (SUVmax) of 11.21. FDG: fluorodeoxyglucose

Contrast orbital computed tomography (CT) demonstrated a well-circumscribed, solid extraconal mass centered around the medial rectus muscle in the left orbit (Figures 2A-2C). The lesion displaced adjacent structures but showed no evidence of bony erosion.

**Figure 3:**
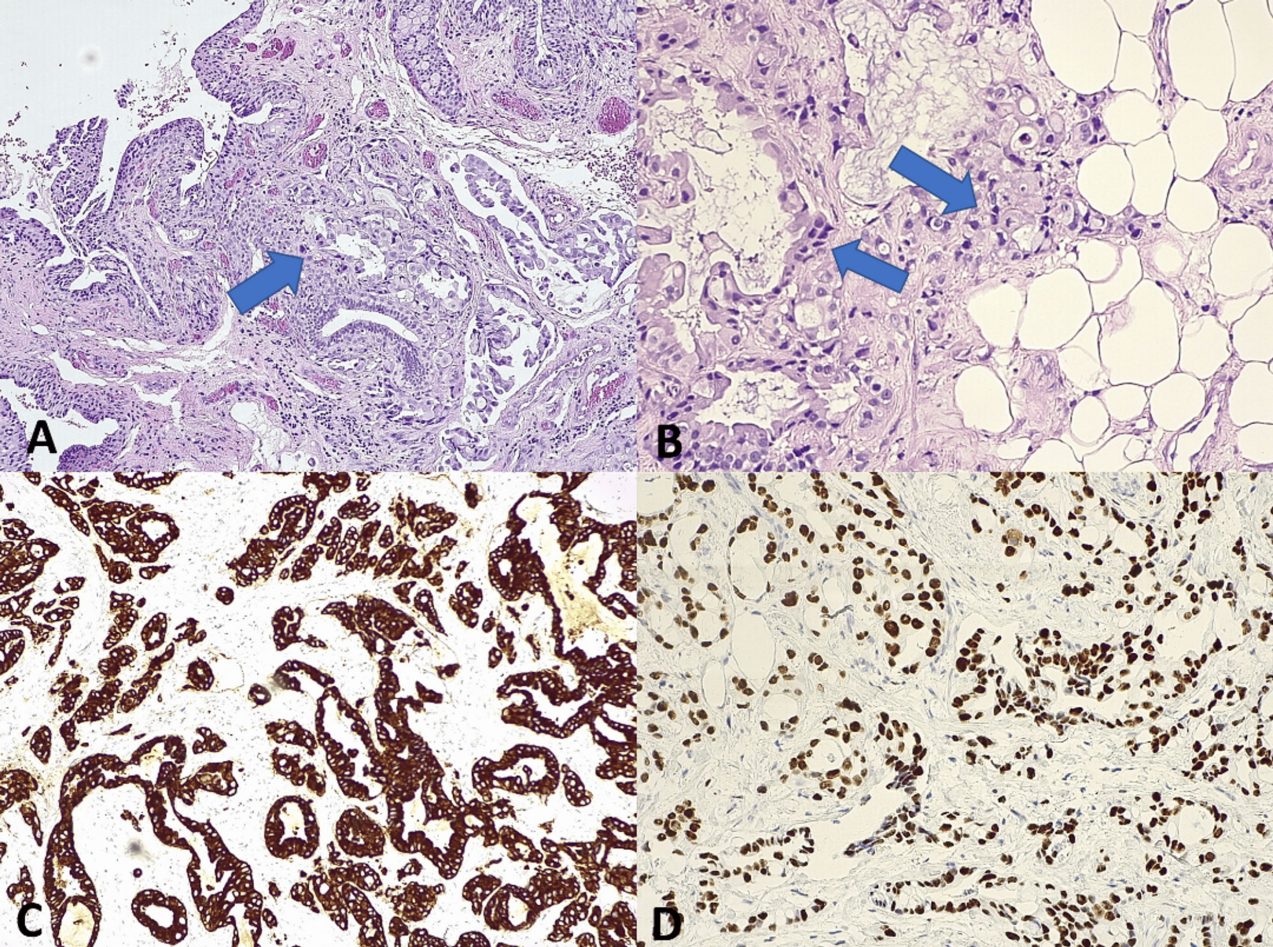
Histopathological examinations. Hematoxylin and eosin stain, ×10. Orbital tissue is infiltrated by malignant epithelial cells with signet-ring morphology (arrow), embedded in abundant mucinous stroma (A). Hematoxylin and eosin stain, ×20. Orbital adipose tissue showing infiltration by neoplastic signet-ring cells within mucinous pools (arrows) (B). Immunohistochemistry for cytokeratin 7 (CK7), ×10. Tumor cells exhibit diffuse cytoplasmic positivity, confirming epithelial origin (C). Immunohistochemistry for p53, ×20. Tumor cells show strong nuclear immunoreactivity, consistent with high-grade malignancy (D).

An exploratory orbitotomy with incisional biopsy was performed. Intraoperatively, a firm, infiltrative mass was observed, involving the globe and encasing the optic nerve. Histopathological examination revealed an atypical epithelial neoplasm composed of irregular glands, cords, nests, and individual cells with enlarged, hyperchromatic nuclei and scant cytoplasm. Occasional signet ring-like cells with intracytoplasmic vacuoles were present. Abundant extracellular mucin and frequent lymphovascular invasion were also observed (Figures 3A, 3B).

Immunohistochemical staining showed strong diffuse positivity for cytokeratin 7 (CK7) and p53, along with weak and patchy expression of CK20 and carcinoembryonic antigen (CEA), findings that supported a primary orbital origin (Figures 3C, 3D). A thorough metastatic workup, including contrast-enhanced thoracoabdominopelvic CT, mammography, salivary gland ultrasonography, and colonoscopy, revealed no evidence of a primary tumor elsewhere.

Despite local surgical intervention, follow-up positron emission tomography (PET) imaging demonstrated rapid systemic dissemination, with the most intense uptake in the left superior nasal orbital region (Figure 2D), consistent with the orbital mass as the dominant lesion. The patient’s condition deteriorated rapidly, and he died six weeks after the initial diagnosis.

## Discussion

Primary mucinous adenocarcinoma of the orbit is an exceedingly rare epithelial malignancy with poorly understood pathogenesis and risk factors [1]. While mucinous adenocarcinomas commonly arise in mucin-secreting tissues such as the gastrointestinal tract, breast, ovary, and skin, their occurrence in the orbit presents significant diagnostic challenges due to clinical and radiological overlap with more prevalent orbital neoplasms, particularly metastatic lesions [1]. To our knowledge, to date, only six cases of metastatic mucinous adenocarcinoma involving the orbit have been reported, five of which originated from gastrointestinal primaries, including rectal, esophageal, gastric, and pancreatic carcinomas [1-5]. The sixth case was an orbital recurrence from a primary mucinous carcinoma of the sweat glands of the eyelid [6]. Moreover, only two cases of primary mucinous adenocarcinoma arising intrinsically within the orbit have been documented [5-7].

Histologically, mucinous adenocarcinomas are characterized by clusters of atypical epithelial cells suspended within abundant extracellular mucin, often forming glandular or cribriform architectures. However, these features are not pathognomonic for primary tumors and are frequently observed in metastases, particularly from breast and gastrointestinal adenocarcinomas. Therefore, immunohistochemical (IHC) analysis is indispensable to differentiate primary from secondary lesions [8,9]. Cytokeratin profiling, especially CK7 and CK20 expression, plays a pivotal role; primary orbital mucinous adenocarcinomas generally exhibit a CK7-positive and CK20-negative immunophenotype, contrasting with gastrointestinal adenocarcinomas, which typically show the opposite pattern [10]. Additionally, IHC markers, including epithelial membrane antigen, CEA, gross cystic disease fluid protein-15, and hormone receptors (estrogen and progesterone), may further aid diagnosis. In our case, the CK7+/CK20- profile combined with a comprehensive negative systemic workup, including thoracoabdominopelvic CT, colonoscopy, and PET scan, strongly supported the diagnosis of primary orbital mucinous adenocarcinoma. The strong nuclear immunoreactivity for p53 observed in our case supports the diagnosis of a high-grade malignancy. Overexpression of p53 is commonly associated with increased tumor aggressiveness and poor prognosis in various epithelial neoplasms, including mucinous adenocarcinomas. Although p53 staining is not specific, its diffuse and intense nuclear positivity in this context reinforces the malignant nature of the lesion and suggests the potential for aggressive clinical behavior [8,9].

Nonetheless, the rapid onset of systemic metastases soon after diagnosis raises the possibility of an occult extraorbital primary tumor undetectable at initial evaluation. This underscores the necessity for vigilant follow-up and repeated systemic reassessment in similar cases. Histopathological examination revealed atypical gland-forming epithelial cells embedded in mucin pools, exhibiting mild to moderate nuclear pleomorphism and occasional mitoses.

Imaging modalities such as CT and MRI are generally nonspecific, often revealing a well-demarcated or infiltrative orbital mass with contrast enhancement and displacement of adjacent structures. These radiologic features can mimic a variety of primary and secondary orbital tumors, further complicating diagnostic efforts. Given its aggressive clinical behavior and reported recurrence rates reaching up to 40% [8], wide local excision remains the cornerstone of treatment. In cases of extensive disease, orbital exenteration may be necessary to achieve complete resection. Adjuvant radiotherapy has been employed to reduce recurrence risk, although its impact on overall survival remains uncertain.

In our patient, despite prompt local management, rapid disease progression with systemic metastases dominated the clinical course. The patient’s deteriorating general condition precluded further therapeutic interventions, culminating in a poor prognosis.

## Conclusions

This case underscores the importance of considering primary mucinous adenocarcinoma in the differential diagnosis of atypical orbital masses, particularly those exhibiting rapid progression. Prompt biopsy alongside comprehensive histopathological and immunohistochemical evaluation is essential for accurate diagnosis. A thorough metastatic workup is imperative even in the absence of clinical signs suggestive of systemic disease, as identifying a primary source critically informs therapeutic decisions and prognosis. Although rare, primary mucinous adenocarcinoma of the orbit may exhibit aggressive behavior and unfavorable outcomes, warranting heightened clinical vigilance and a multidisciplinary management approach.
